# First Dinosaur Tracks from the Arabian Peninsula

**DOI:** 10.1371/journal.pone.0002243

**Published:** 2008-05-21

**Authors:** Anne S. Schulp, Mohammed Al-Wosabi, Nancy J. Stevens

**Affiliations:** 1 Natuurhistorisch Museum Maastricht, Maastricht, The Netherlands; 2 Department of Geology, Sana'a University, Sana'a, Republic of Yemen; 3 Department of Biomedical Sciences, College of Osteopathic Medicine, Ohio University, Athens, Ohio, United States of America; Paleontological Institute, Russian Federation

## Abstract

**Background:**

The evolutionary history of Mesozoic terrestrial vertebrates from the Arabian Peninsula is virtually unknown. Despite vast exposures of rocky outcrops, only a handful of fossils have yet been described from the region. Here we report a multi-taxon dinosaur track assemblage near Madar village, 47 km north of Sana'a, Republic of Yemen. This represents the first dinosaur tracksite from the Arabian Peninsula, and the only multi-taxon dinosaur ichnosite in the Middle East.

**Methodology/Findings:**

Measurements were taken directly from trackway impressions, following standard ichnological conventions. The presence of bipedal trackmakers is evidenced by a long series of pes imprints preserving smoothly rounded posterior margins, no evidence of a hallux, bluntly rounded digit tips and digital divarication angles characteristic of ornithopod dinosaurs. Nearby, eleven parallel quadrupedal trackways document a sauropod herd that included large and small individuals traveling together. Based on the morphology of manus impressions along with a narrow-gauged stance, the quadrupedal trackways were made by non-titanosauriform neosauropods. Additional isolated tracks and trackways of sauropod and ornithopod dinosaurs are preserved nearby.

**Conclusions/Significance:**

Taken together, these discoveries present the most evocative window to date into the evolutionary history of dinosaurs of the Arabian Peninsula. Given the limited Mesozoic terrestrial record from the region, this discovery is of both temporal and geographic significance, and massive exposures of similarly-aged outcrops nearby offer great promise for future discoveries.

## Introduction

The dinosaur record of the Arabian Peninsula is limited to reports of isolated axial elements from the Sultanate of Oman [1], [2], and sauropod remains of possibly Jurassic age from Yemen [3]. More broadly, published reports from the region are restricted to a distal tibia of an indeterminate theropod from Syria [4], a fragmentary ornithopod appendicular specimen from the Late Cretaceous of Jordan [5], and two brachiosaurid teeth from the early Cretaceous of southern Lebanon [6]. Finally, theropod tracks have been reported near Jerusalem [7]–[9]. Here we describe a multi-taxon dinosaur ichnosite from the Republic of Yemen, representing the first record of dinosaur trackways on the Arabian Peninsula.

### Locality

**Figure 1:**
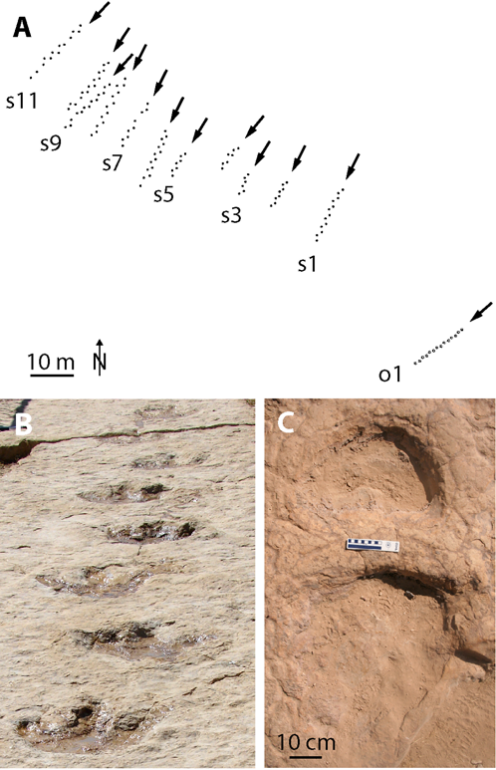
Dinosaur trackways from Arhab locality, Republic of Yemen. (A)-Map of tracksite with ornithopod (trackway o1) and sauropod (trackways s1–s11) trackways, (B)-Trackway of the ornithopod (trackway o1: steps 3–10), and (C)-Sauropod left manus and pes print (trackway s6: step 12).

The dinosaur trackways described herein are located near the village of Madar, Arhab area, 47 km north of Sana'a, the capitol of the Republic of Yemen [Fig. 1A]. The main site, at 15°46′49″N, 44°14′25″E, is approximately 3 km west of the main road, and has been signposted and fenced by the Yemen Geological Survey. Additional tracks have been recognized nearby, within the villages of Arhab and Bait al Washr.

### History of discovery

The first set of tracks at Arhab was discovered by Mohammed Al-Daheri, a local journalist. He notified M.A.W., who worked with the Yemen Geological Survey to protect the locality and contacted A.S. and N.J.S. to collaboratively assemble a detailed description of the tracksite. During the mapping effort, additional trackways were discovered west of the initial locality, including two poorly preserved trackways of bipeds along with 11 relatively well-preserved subparallel quadrupedal series [Fig 1]. Prospecting and mapping in December 2006 and February 2007 yielded two additional track sites at Bait al Washr village [15°48′41″N, 44°15′52″E], some 6 km east of the main track locality, as well as a few poorly preserved trackways preserved in the bedrock within the boundaries of the village.

### Geological setting

The Arhab dinosaur tracksite is situated in a sub-horizontal outcrop of yellow limestones, locally covered with basalt flows. The track-bearing beds, representing a coastal mudflat environment, are of Middle Jurassic to lowermost Cretaceous age. The strata were initially introduced as Amran Series [10], and further described and defined as Amran Group [11]. The Amran Group has subsequently been dated as Callovian-Berriasian in age based on ammonite fauna [12]; foraminiferal assemblages suggest a Bathonian-Berriasian age [13]. The track-bearing layers are predominantly exposed as dipslopes, with little vegetation cover, and extending over a much wider area than actually prospected during the brief fieldwork, underscoring the potential for recognizing an even more diverse ichnofauna in the region.

## Results

### Identification of the bipedal trackmaker

The first tracksite discovery, a bipedal tridactyl series spanning 14 m, reveals 15 consecutive tracks heading in a SSW direction [Fig. 1A, B]. Pes length and width each average 56 cm. Average step and stride lengths are 107 cm and 207 cm, respectively. Corresponding average pace angulation is 151°. On the relatively well-preserved first three tracks, the axis of digit III exhibits an outward rotation of 4° from the trackway midline.

We refer these bipedal tracks to ornithopod trackmakers based on the following characteristics [14], [15]: tracks are approximately equal in length and width; digits [particularly digit III] preserve a u-shaped outline with bluntly rounded tips; digit III width/length ratio is >0.5; digits exhibit no curvature nor is there evidence of a hallucal impression; the divarication of digit II-IV averages 65°; and the rear edge of footprint exhibits a smooth, convex margin.

### Identification of the quadrupedal trackmaker

West of the tridactyl trackway, 11 subparallel quadrupedal trackways [Fig. 1A, C] preserve evidence of large and small quadrupedal animals traveling together in a herd. The longest [16 m] of these trackways currently preserves 16 consecutive footprints. As with the bipedal trackway, potential exists for discovering additional tracks by further exposing the layer along the northern edge of the site. Tracks do not preserve distinctive evidence of claws on either the manus or the pes [Fig. 1C]. Average stride lengths of the 11 individuals range from 187–252 cm; average pace angulation is 113°; pes length from 43–70 cm. Well-preserved tracks for the 11 individuals indicate average pes dimensions of 57 cm in length by 46 cm in width. Manus outlines are distinct for fewer trackmakers, but range between 21–31 cm in length and 34–42 cm in width.

We refer these quadrupedal tracks to neosauropods based on the following characteristics: the anteroposteriorly short, u-shaped manus impressions suggest an arc-shaped articulation of metacarpals, as observed in the Neosauropoda [16]. The quadrupedal trackways at Arhab are relatively narrow-gauge, with the left and right pes tracks touching [but not overlapping] the trackway midline, unlike the wide-gauge trackways typically associated with titanosauriforms [16]. Given this narrow-gauge stance, together with a more derived, arc-shaped manus impression, quadrupedal tracks at Arhab were likely made by nontitanosauriform neosauropods.

## Discussion

Co-occurrence of sauropod and ornithopod tracks in carbonates is relatively uncommon. The presence of neosauropod and ornithopod trackways in Yemen is consistent with the presence of body fossils of both groups on the African continent by the late Jurassic [17]. At that time, the African and Arabian Peninsular landmasses had not yet been separated by the Red Sea.

Early potential ornithopods are known from the early Jurassic of South Africa, e.g., heterodontosaurs [18], [19], with undisputed ornithopods subsequently known throughout Africa, Asia, Europe, North America and Australia, reaching a zenith of known diversity [17] during the Cretaceous. Early Cretaceous bipedal dinosaur tracks have been reported from Cameroon [20]; with the 39 cm long tridactyl prints having a morphology “consistent with either iguanodont or theropod morphology” [20: p. 350]. As ornithopod body fossils are already well-represented in Africa by the late Jurassic [21]–[23], their presence in Yemen is not unexpected. Because ornithopod tracks of this size are not well known from the late Jurassic, these tracks may suggest an early Cretaceous age for the deposits. If not, the ichnosite is of additional importance in preserving evidence of a rare early occurrence of a large ornithopod taxon.

The earliest sauropods are described from Upper Triassic of South Africa [24], indicating that the initial diversification of the group likely occurred before the separation of much of Pangaea, and as with the ornithopods, certainly predated the breakup of Gondwana. By the mid-Jurassic, sauropod dinosaurs are known from either body fossils or footprints from every major continental landmass except Antarctica [25], indicating a more or less global distribution. Many sauropods from the southern landmasses belong to the Titanosauriformes [26]. Titanosauriforms are characterized by derived postcranial morphological features including medially deflected femoral shafts and laterally expansive ilia that have been associated with a wide-gauge stance [27]–[29]. In contrast, the trackways at Arhab are relatively narrow-gauge, with the left and right pes tracks touching [but not overlapping] the trackway midline. Together, the narrow-gauge stance along with the more derived, arc-shaped manus impressions suggests the presence of nontitanosauriform neosauropods. Further, the presence of parallel sauropod trackways at Arhab has implications for inferring the behavior of the trackmakers, as the 11 trackways are roughly parallel, evenly spaced, and estimates of speed based on stride lengths appear fairly constant at approximately 3km/hour despite a likely fourfold difference in body mass between the smallest and largest individuals. Neosauropod taxa are well documented from the Gondwanan landmasses, and African neosauropods of consistent size with the trackmakers at Arhab include *Nigersaurus*, *Rebbachisaurus* and *Algoasaurus* [17]. Finally, narrow-gauge sauropod trackways have been reported from Morocco [30], [31] and Zimbabwe [32].

If ichnofossils are to be used as independent data points, their taxonomic attribution should not be based solely upon spatial and temporal coincidence [25]. The footprints described herein preserve sufficient morphological detail to support the presence of both ornithopod and sauropod dinosaurs at Arhab. Taken together with the approximate age of the deposits, this assessment accords well with global patterns in dinosaurian evolution. Moreno and Benton [33] review what appears to be a marked transition from sauropod-dominated faunas, to ornithopod-dominated faunas at the Jurassic-Cretaceous boundary. It is only with intensified sampling effort that such patterns can be explored and tested in Afro-Arabia.

## Materials and Methods

Trackways were exposed by clearing away sand, small rocks and debris. From the impressions, we obtained the following measurements: manus length and width (defined respectively as the maximum antero-posterior and mediolateral dimensions of an individual manus impression), pes length and width (defined respectively as the maximum antero-posterior and mediolateral dimensions of an individual pes impression), stride length (defined as the distance between the posteriormost margins of consecutive tracks of the same limb), step length (defined as the distance between the posteriormost margins of consecutive tracks within a forelimb pair or hind limb pair), pace angulation (defined as the angle formed between line segments connecting the posteriormost margins of consecutive hind limb tracks on ornithopod tracks, and the angle formed between line segments connecting the better-preserved anteriormost point of the pes on sauropod tracks), and digit divarication (defined as the angle formed between line segments approximating the long axes of digits II and IV). Estimates of speed for the 11 sauropod trackmakers were made using pes lengths and stride lengths measured directly from the tracksite, following calculations outlined in Alexander (1976): *v*≈0.25*g* ^0.5^ *SL* ^1.67^ *h* ^−1.17^, where *v* indicates velocity, *g* equals the gravitational constant, *SL* equals stride length, and *h* equals hip height. Impressions with obvious overlap or breakage were excluded from the analysis. Individual tracks were traced onto transparent plastic film overlays. Overlays were scanned into Adobe Illustrator (version 10) and field measurements of pace angulation were checked for accuracy using Spot Advanced (version 3.5).
